# Analysis of the Sensing Properties of a Highly Stable and Reproducible Ozone Gas Sensor Based on Amorphous In-Ga-Zn-O Thin Film

**DOI:** 10.3390/s18010163

**Published:** 2018-01-09

**Authors:** Chiu-Hsien Wu, Guo-Jhen Jiang, Kai-Wei Chang, Zu-Yin Deng, Yu-Ning Li, Kuen-Lin Chen, Chien-Chung Jeng

**Affiliations:** 1 Institute of Nanoscience, National Chung Hsing University, Taichung 402, Taiwan; flame0350@hotmail.com (G.-J.J.); sam519006@gmail.com (K.-W.C.); g103054104@mail.nchu.edu.tw (Z.-Y.D.); g105017008@mail.nchu.edu.tw (Y.N.L.); ccjeng@phys.nchu.edu.tw (C.-C.J.); 2 Department of Physics, National Chung Hsing University, Taichung 402, Taiwan; klchen@phys.nchu.edu.tw

**Keywords:** IGZO, ozone sensor, reproducibility, ppb-level ozone

## Abstract

In this study, the sensing properties of an amorphous indium gallium zinc oxide (a-IGZO) thin film at ozone concentrations from 500 to 5 ppm were investigated. The a-IGZO thin film showed very good reproducibility and stability over three test cycles. The ozone concentration of 60–70 ppb also showed a good response. The resistance change (Δ*R*) and sensitivity (*S*) were linearly dependent on the ozone concentration. The response time (*T*_90-res_), recovery time (*T*_90-rec_), and time constant (τ) showed first-order exponential decay with increasing ozone concentration. The resistance–time curve shows that the maximum resistance change rate (dRg/dt) is proportional to the ozone concentration during the adsorption. The results also show that it is better to sense rapidly and stably at a low ozone concentration using a high light intensity. The ozone concentration can be derived from the resistance change, sensitivity, response time, time constant (τ), and first derivative function of resistance. However, the time of the first derivative function of resistance is shorter than other parameters. The results show that a-IGZO thin films and the first-order differentiation method are promising candidates for use as ozone sensors for practical applications.

## 1. Introduction 

Gas sensors are widely used in several environmental and industrial applications. Good gas sensors should possess several characteristics, including high sensitivity, fast response, low energy consumption, long-term operation capability, and low fabrication cost [1,2,3,4,5,6]. Metal oxide semiconductors (MOSs) are the most popular gas-sensing materials, because they have low fabrication costs, high sensitivities, and long-term operation capability. Thin films of MOSs such as tin oxide and zinc oxide, 2D materials, and graphene are widely used in different gas sensors to detect diverse target materials [1,2,3,4,5,6,7]. Recently, low-melting liquid metal-based reaction environments have been used to synthesize oxide nanomaterials with low dimensionality (2D materials) [8,9]. The thin oxide layer is a suitable candidate for sensing gases such as NO_2_, CH_4_ and NH_3_ [10].

We previously reported that the MOS amorphous indium-gallium-zinc oxide (InGaZnO_4_, a-IGZO) has potential as an excellent ozone gas sensor [11,12]. IGZO is an *n*-type semiconductor with a wide band gap (3.2–3.5 eV). It has extremely high transmittance in the visible range and large electron mobility. IGZO is used in the display industry, for example, in 4K-resolution low-power IGZO LCD panels [13,14,15,16]. It is also used in gas sensors for different targets [17,18,19].

In this study, the relation between different parameters and ozone concentration was established. The stability and reproducibility of the films exposed to ozone is reported. The changes in IGZO film sensitivity, response time, time constant, and the rate of resistance change relative to the ozone concentration in the range of 500 ppb–2 ppm were evaluated. Fast, stable methods for sensing ozone are also discussed.

## 2. Experimental

*n*-type transparent IGZO thin films (thickness = 60–70 nm) were deposited onto a 10 × 10 mm^2^ glass substrate using an RF sputtering system. The IGZO ceramic target had an atomic ratio of In: Ga:Zn = 1:1:1. The films were deposited at room temperature and annealed at 100 °C for 1 h. The deposition pressure of pure Ar gas was 200 mTorr. The RF power was 100 W. The crystallinity and morphology of the films were studied via XRD analysis; the results showed that the films were amorphous. The figure is shown in our previous reports [11,12].

A home-made test chamber was built to house both the a-IGZO films and UV LED. The thin films were continuously irradiated using a 365-nm UV LED (P5-40-B, SemiLEDs), and the electrical properties were measured at room temperature (25 °C, RH = ~30%). The IGZO thin films were continuously irradiated using a 365-nm UV LED at room temperature. A UV LED was used as the source of excited electrons. The intensity of the UV LED was fixed at 13.92 mW/cm^2^. Ozone was generated from an ozone generator and passed through a low-flow metering valve. Then, the ozone was mixed with dry air obtained from an air pump before channeling into the test chamber. Ozone was initially sensed by the IGZO sensor. The ozone concentration inside the chamber was also monitored using a commercial ozone monitor (2B Tech., Model-106L). The ozone concentrations reported in this study were measured using the commercial ozone monitor. The details of the experimental schematics and processes have been reported in our previous study [11,12,20,21].

## 3. Results and Discussion

The quality of the films analyzed via XRD, AFM, and UV-visible spectroscopy has been reported in previous studies [11,12]. 

**Figure 1:**
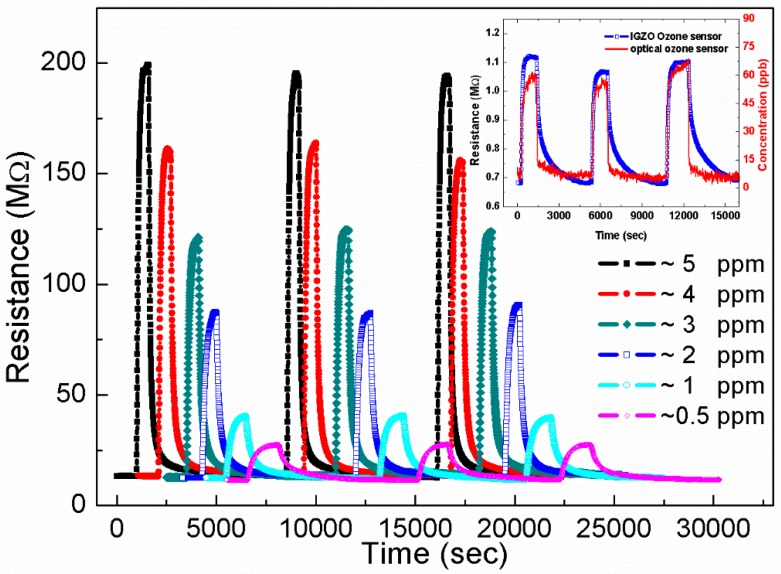
The resistance-time (*R*-*T*) curves of an IGZO film exposed to ozone concentrations from 500 to 5 ppm. The inset show another sample under the concentration of 60–70 ppb.

Figure 1 shows the resistance-time (*R*-*T*) curves of an IGZO film exposed to ozone concentrations from 500 to 5 ppm with a light intensity of 13.92 mW/cm^2^. To clearly observe the *R*-*T* curves, the time axis was shifted for the samples with 0.5–4-ppm exposure [4,22,23,24,25,26,27].

The lowest resistance (*R_a_* in air) and saturation resistance (*R_g_* in gas) of the film are almost identical over the course of three exposure cycles at the same ozone concentration. The excellent reproducibility of the *R*-*T* curves shows that the IGZO films have good performance as ozone gas sensors. The resistances were stabilized at different ozone concentrations.

When the measurements were performed under atmospheric conditions, the response equations can be expressed as follows:

(1) $$h\nu \to h^{+}+e^{-}(\mathrm{photoexcitation})$$

(2) $$O_{3}+e^{-}\to O_{3}^{-}(\mathrm{adsorption})$$

(3) $$O_{3}^{-}+h\nu \to O_{2(g)}^{-}+e^{-}(\mathrm{desorption})$$

The ozone molecules were adsorbed on the surface when they were channeled. The electrons were trapped by ozone, increasing the resistance. *R_g_* is dependent on ozone concentration.

**Figure 2:**
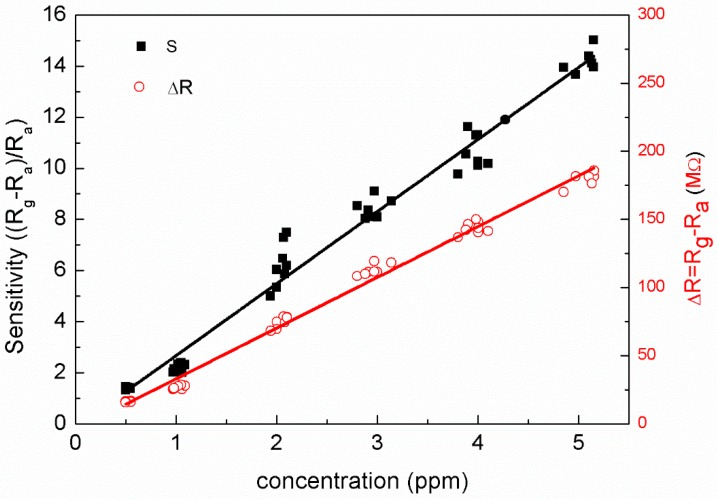
The relation between ozone concentration and both resistance change and sensitivity.

Figure 2 shows the relation between ozone concentration and both resistance change (Δ*R* = *R_g_* − *R_a_*) and sensitivity (*S* = (*R_g_* − *R_a_*)/*R_a_* = Δ*R*/*R_a_*). Both Δ*R* and *S* linearly increase with increasing ozone concentration. The magnitude of Δ*R* clearly differs at different ozone concentrations. The values of Δ*R* were 17 MΩ and 175 MΩ at ~0.5 ppm and ~5 ppm, respectively, while *S* increased from 1.4 to 14 across the same concentration range. Both the results indicate an order of magnitude increase in the measured parameter. The Δ*R* values had less variation at a given ozone concentration. These results indicate that when the ozone concentration increases, more ozone molecules are adsorbed onto the surface of the film [3,4]. According to the plot, the limit of detection is approximately 80 ppb [28]. The inset of Figure 1 shows the *R*-*T* plot of another sample at a concentration of 60–70 ppb; the IGZO thin film has potential for sensing ozone below 100 ppb. The ozone concentrations of 60–70 ppb were measured using the commercial ozone monitor. This sensing range of ozone concentration is good for practical applications (Ozone concentration over 100 ppb is harmful to health).

**Figure 3:**
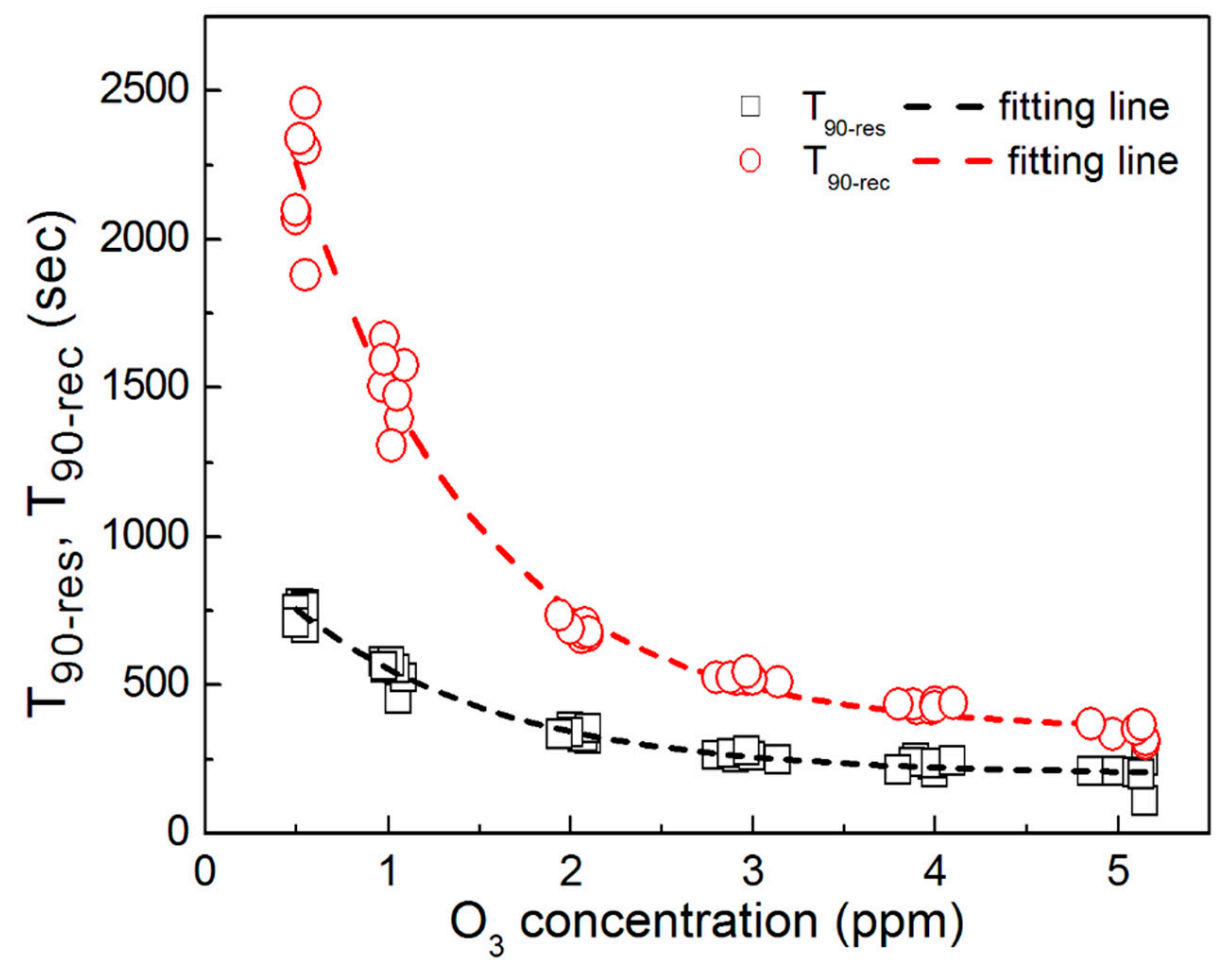
The response (*T*_90-res_) and recovery (*T*_90-rec_) times of the gas sensor at several different ozone concentrations.

Figure 3 shows the response (*T*_90-res_) and recovery (*T*_90-rec_) times of the gas sensor at several ozone concentrations calculated from the results shown in Figure 1. *T*_90-res_ is defined as the time required to reach 90% of Δ*R*, whereas *T*_90-rec_ is the time required for resistance to recover 90% of Δ*R*. For a low ozone concentration of 0.5 ppm, *T*_90-res_ was between 690 and 775 s, and *T*_90-rec_ was between 1885 and 2470 s. At higher concentrations of 3-, 4- and 5-ppm ozone, the *T*_90-res_ values were approximately 270, 230 and 210 s, respectively, and the *T*_90-rec_ values were between 360 and 530 s [25]. Both the response and recovery times show a first-order exponential decay with increasing ozone concentration in contrast to the linear relation between the Δ*R* and ozone concentrations. For the same treatment, *T*_90-res_ was always shorter than *T*_90-rec_. It was also found that *T*_90-res_ and *T*_90-rec_ determined at low concentrations have a larger variance than those determined at high concentrations; however, *R_a_*, *R_g_* and Δ*R* are almost identical, possibly because of an unstable ozone flow at low concentrations. In this study, the samples were exposed to the same light intensity under continuous irradiation. The absorption and desorption processes are in dynamic equilibrium. The desorption particles are almost the same at both low and high gas concentrations at the same light intensity. The total resistance increases when R_ad-Ozone_ (adsorption resistance) exceeds R_de-Ozone_ (desorption resistance). The R_de-Ozone_ is far less than R_ad-Ozone_ at the high concentration. Therefore, the response and recovery times might have decreased as the gas concentration increased.

**Figure 4:**
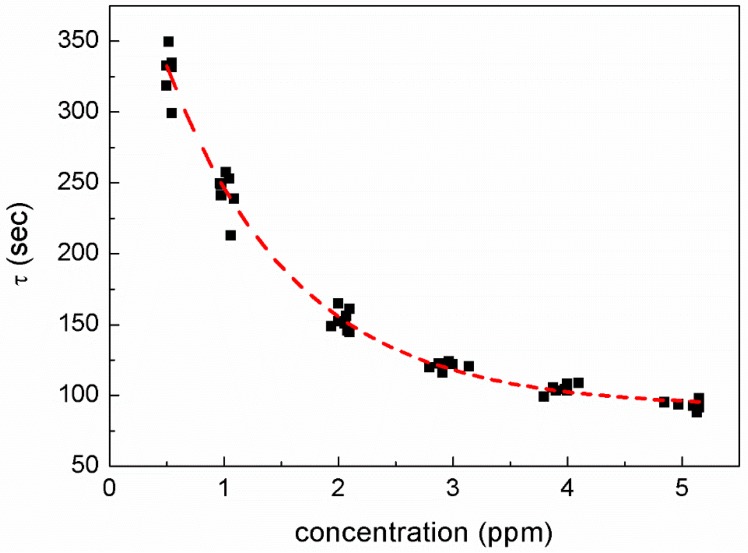
A plot of time constant as a function of O_3_ concentration.

The *R*-*T* relation can be fitted using the equation, where A and B are constant, t is the time taken for ozone channeling (s), and τ is the time constant of absorption. The behavior of ozone that adsorbs onto an IGZO film is probably similar to the charging of a capacitor. Figure 4 shows a plot of τ as a function of ozone concentration. The results show that the τ value decreased as the ozone concentration increased. Similar to the *T*_90-res_ and *T*_90-rec_ values, the range of τ values recorded at a low ozone concentration (τ = 324 ± 25 s at 0.5 ppm) is much wider than that recorded at a high ozone concentration (5 ppm), where τ is ~90 ± 5 s. No significant change in gas response was observed in the range 3–5 ppm; however, at low gas concentrations, large variances were observed in the sensing properties of the film.

Based on the above results, the corresponding resistance at the surface of IGZO as a function of time during the adsorption and desorption of an oxidizing gas can be described via the following equations:

(4) $$R_{g}(t)=R_{0}-\Delta R\;\exp\left[\frac{-t}{\tau_{abs}}\right]$$

(5) $$R_{g}(t)=R_{2}+\Delta R\;\exp\left[\frac{-t}{\tau_{des}}\right]$$

where *R*(*t*) is the resistances after the gas exposure; *R*_0_ is a constant; Δ*R* is the difference between the initial and saturated resistance (*R_g_* − *R_a_*); *t* is the time taken for ozone channeling (s); and *τ_ab__s_* and *τ_des_* are the adsorption and desorption time constants (s), respectively.

Differentiating Equations (1)–(4), respectively, are obtained as follows:

(6) $$\frac{dR_{g}(t)}{dt}=\frac{\Delta R}{\tau_{abs}}\exp\left[-\frac{t}{\tau_{abs}}\right]$$

(7) $$\frac{dR_{g}(t)}{dt}=\frac{-\Delta R}{\tau_{des}}\exp\left[-\frac{t}{\tau_{des}}\right]$$

According to Equation (3), the maximum rate of change is Δ*R*/*τ_abs_* during absorption.

(8) $$(\frac{dR_{g}(t)}{dt})\propto \frac{\Delta R}{\tau_{des}}\propto \frac{aC}{be^{-kC}}\propto \frac{aC}{b(1-kC+1/2{\left(kC\right)}^{2}+.....)}\propto \frac{a}{b}C$$

where *a*, *b* and *k* are constants. Because the *C* is in the ppb or ppm level, the value of *kC* is much less than 1. The maximum value of *dR_g_*(*t*)/*dt* is directly proportional to and dependent on the ozone concentration during the absorption. The maximum rate of change is −Δ*R*/*τ_des_* during the desorption. In addition, *dR_g_*(*t*)/*dt* is negative and negatively associated with ozone concentration.

**Figure 5:**
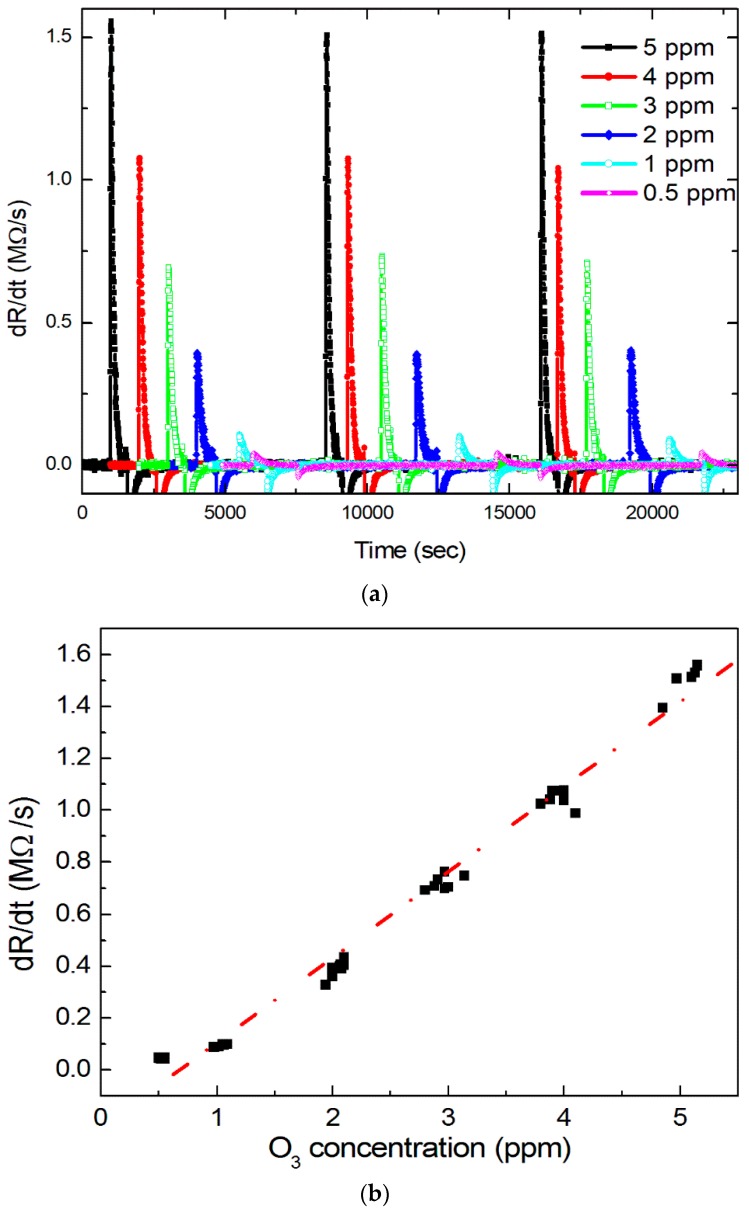
(**a**) The resistance change rate-time relation; (**b**). The maximum value of the first derivative of the *R*-*T* curves given in Figure 1.

Figure 5a shows the resistance change rate (*dR_g_*(*t*)/*dt*)-time relation. The rate of change in resistance is dependent on the ozone concentration [21]. At higher gas concentrations, more ozone molecules were adsorbed on the film, and the film lost electrons rapidly. Therefore, a high ozone concentration increases the amount of resistance change.

Figure 5b shows the maximum value of the first derivative of the *R*-*T* curves shown in Figure 1. The correlation between the maximum value of the resistance change rate and ozone concentration is linear and highly positive, indicating that the rate of resistance change is large at high ozone concentrations. The maximum *dR_g_*(*t*)/*dt* linearly increased with increasing ozone concentration. The results agree with Equation (8). At high ozone concentrations (2–5 ppm), the maximum values of *dR_g_*(*t*)/*dt* change significantly (between 0.36 and 1.5 MΩ/s). In the low concentration range (0.5–1 ppm), the maximum values of d *Rg*(*t*)/*dt* were between 45 and 90 KΩ/s; this difference is of considerable size, and can be easily distinguished. Our resistance meter can detect several ohms; therefore, it is able to sense levels of ozone of several ppb.

**Figure 6:**
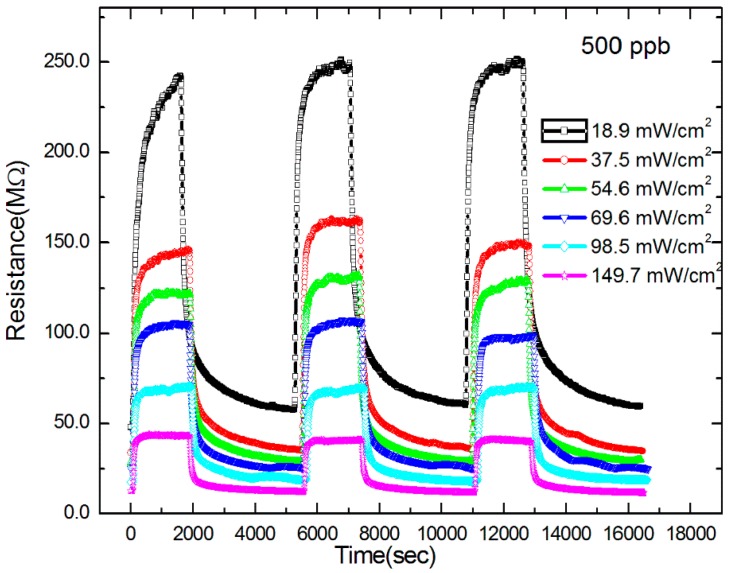
A 13-nm-thick IGZO sensor under different light intensities at 500-ppb O_3_.

Figure 6 shows the *R*-*T* curves of a 13-nm-thick IGZO sensor under different light intensities at 500-ppb ozone. The sample was irradiated for three test cycles with light intensities in the range 18.9–149.7 mW/cm^2^. The *R_a_*, *R_g_* and Δ*R* values were different for different light intensities at the same ozone concentration. At a low-intensity light of 18.9 mW/cm^2^, Δ*R* was ~200 MΩ, and *T*_90-res_ was ~475 s. At a higher light intensity of 149.7 mW/cm^2^, Δ*R* was ~30 MΩ, and *T*_90-res_ was ~110 s. *R_a_*, *R_g_* and Δ*R* had slight variances across the three test cycles. The samples provided highly reproducible results at all the investigated light intensities, even though the sensors provided slightly more reproducible results under high-intensity light than under low-intensity light.

**Figure 7:**
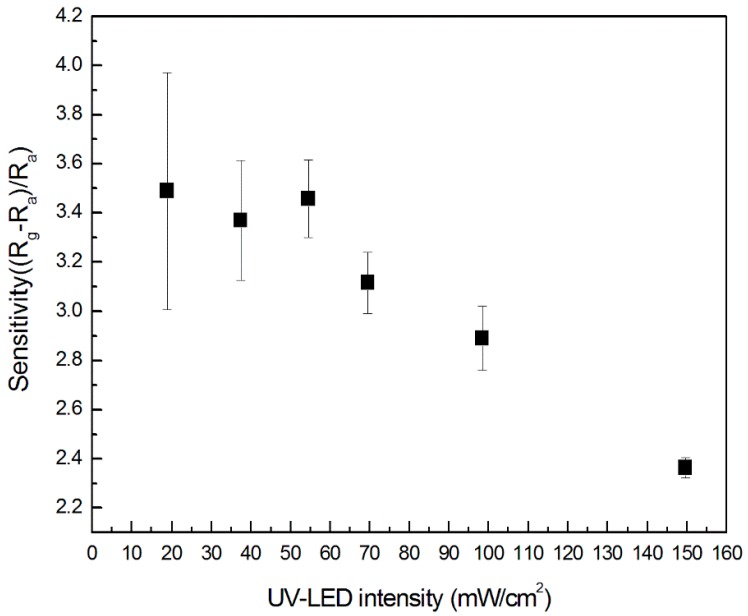
The relation between IGZO sensor sensitivity and light intensity.

This is probably because the dynamic equilibrium (i.e., the adsorption and desorption of gas molecules from the IGZO sensor) is rapid and stable under high-intensity light. Figure 7 shows the relation between sensor sensitivity and light intensity. The sensitivity was obtained from Figure 6. The variance in sensitivity at low intensity was larger than that at high intensity, at a low ozone concentration of 500 ppb.

## 4. Conclusions

An IGZO ozone sensor successfully detected ozone concentrations ranging from 500 to 5 ppm. The results show good consistency for the recorded values of *R_a_*, *R_g_* and Δ*R*. Both Δ*R* and *S* exhibited a linear dependence on ozone concentration. *T*_90-res_, *T*_90-rec_ and τ showed first-order exponential decay with increasing ozone concentration. The rate of resistance change linearly increased with increasing ozone concentration. The maximum value of the first derivative of the resistance curve was also dependent on the ozone concentration. The results show that *dR_g_*/*dt*, *T*_90-res_, *T*_90-rec_ and τ can be used to determine the ozone concentration. For a given ozone concentration, the time of the first derivative function of resistance is shorter than other parameters, including *T*_90-res_, *T*_90-rec_ and τ. In the low-concentration range (0.5 ppm), the maximum values of *dR_g_*/*dt* were between ~45 KΩ/s; this difference is considerable. Therefore, this method can be used to sense ozone at several ppb levels.

The value of Δ*R* recorded under low-intensity light was larger than that recorded under high-intensity light; however, compared with low-intensity light, the time required to reach a stable level of ozone absorption onto the IGZO sensor was reduced in high-intensity light. The variance in sensor sensitivity was higher under low-intensity light than that under high-intensity light. It is better to sense a low ozone concentration using a high-intensity light. Thus, IGZO is a promising candidate for use as an ozone sensor.
